# Autocalibration of accelerometer data for free-living physical activity assessment using local gravity and temperature: an evaluation on four continents

**DOI:** 10.1152/japplphysiol.00421.2014

**Published:** 2014-08-07

**Authors:** Vincent T. van Hees, Zhou Fang, Joss Langford, Felix Assah, Anwar Mohammad, Inacio C. M. da Silva, Michael I. Trenell, Tom White, Nicholas J. Wareham, Søren Brage

**Affiliations:** ^1^MoveLab, Institute of Cellular Medicine, Newcastle University, Newcastle, United Kingdom;; ^2^Department of Statistics, University of Oxford, Oxford, United Kingdom;; ^3^Activinsight, Limited, Kimbolton, United Kingdom;; ^4^University of Yaoundé, Yaoundé, Cameroon;; ^5^Dasman Diabetes Institute, Kuwait City, Kuwait;; ^6^Federal University of Pelotas–Postgraduate Program in Epidemiology, Pelotas, Brazil; and; ^7^Medical Research Council Epidemiology Unit, University of Cambridge, Cambridge, United Kingdom

**Keywords:** calibration, accelerometry, physical activity, epidemiology, GENEActiv

## Abstract

Wearable acceleration sensors are increasingly used for the assessment of free-living physical activity. Acceleration sensor calibration is a potential source of error. This study aims to describe and evaluate an autocalibration method to minimize calibration error using segments within the free-living records (no extra experiments needed). The autocalibration method entailed the extraction of nonmovement periods in the data, for which the measured vector magnitude should ideally be the gravitational acceleration (1 *g*); this property was used to derive calibration correction factors using an iterative closest-point fitting process. The reduction in calibration error was evaluated in data from four cohorts: UK (*n* = 921), Kuwait (*n* = 120), Cameroon (*n* = 311), and Brazil (*n* = 200). Our method significantly reduced calibration error in all cohorts (*P* < 0.01), ranging from 16.6 to 3.0 m*g* in the Kuwaiti cohort to 76.7 to 8.0 m*g* error in the Brazil cohort. Utilizing temperature sensor data resulted in a small nonsignificant additional improvement (*P* > 0.05). Temperature correction coefficients were highest for the *z*-axis, e.g., 19.6-m*g* offset per 5°C. Further, application of the autocalibration method had a significant impact on typical metrics used for describing human physical activity, e.g., in Brazil average wrist acceleration was 0.2 to 51% lower than uncalibrated values depending on metric selection (*P* < 0.01). The autocalibration method as presented helps reduce the calibration error in wearable acceleration sensor data and improves comparability of physical activity measures across study locations. Temperature ultization seems essential when temperature deviates substantially from the average temperature in the record but not for multiday summary measures.

wearable accelerometers are increasingly used in the assessment of physical activity (2, 4, 6). In recent years accelerometers have become available that are feasible for long-term monitoring of behavior in population studies, while at the same time being capable of storing weeklong data in *g*-units (1 standard *g* = 9.80665 m/s^2^) at a sample frequency high enough to capture the main frequencies of body movement, referred to as raw data accelerometry (12). Population studies collecting raw accelerometer data include surveillance studies like NHANES (26) in the U.S. and national biobanks such as UK Biobank (27).

An acceleration sensor works on the principle that acceleration is captured mechanically and converted into an electrical signal, which depending on the sensor type is either a voltage, a resistance, or a capacitance (13). The relationship between the electrical signal and the acceleration is usually assumed to be linear, involving an offset and a gain factor. We shall refer to the establishment of the offset and gain factor as the sensor calibration procedure (5, 18). Accelerometers are usually calibrated as part of the manufacturing process under nonmovement conditions using the local gravitational acceleration as a reference (5, 18). The manufacturer calibration can later be evaluated by holding each sensor axis parallel (up and down) or perpendicular to the direction of gravity; readings for each axis should be ±1 and 0 *g*, respectively (5, 18).

However, this procedure can be cumbersome in studies with a high throughput. Furthermore, such a calibration check will not be possible for data that have been collected in the past and for which the corresponding accelerometer device does not exist anymore. Techniques have been proposed that can check and correct for calibration error based on the collected triaxial accelerometer data in the participant's daily life without additional experiments, referred to as autocalibration (6a, 8–10, 19). The general principle of these techniques is that a recording of acceleration is screened for nonmovement periods. Next, the moving average over the nonmovement periods is taken from each of the three orthogonal sensor axes and used to generate a three-dimensional ellipsoid representation that should ideally be a sphere with radius 1 *g*, see example in Fig. 1. Here, deviations between the radius of the three-dimensional ellipsoid and 1 *g* (ideal calibration) can then be used to derive correction factors for sensor axis-specific calibration error (6a, 8–10, 19).

**Fig. 1.**
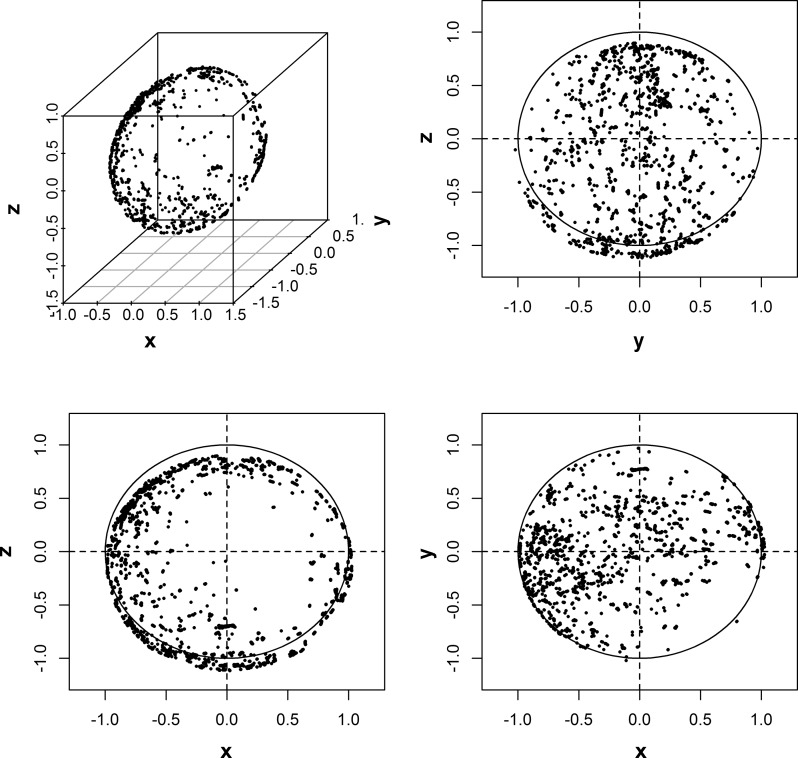
Example 3-dimensional ellipsoidal data based on a 6-day measurement. Also shown are 2-dimensional projections; the circles (radius = 1 *g*) indicate the shape of the data if perfect calibration would apply.

Previously published work on autocalibration techniques focused on the technical description and proof of concept but did not demonstrate feasibility and accuracy in wrist accelerometer data collected under real study conditions, involving participants under free-living conditions (daily life) and in a diverse sample of the global population (6a, 8–10, 19). Furthermore, it remains unknown whether autocalibration has a significant impact on acceleration metrics typically used for physical activity assessment.

Temperature has been identified as a potential source of calibration error in low cost acceleration sensors (20). The specification sheet of the acceleration sensor chip used in the GENEActiv accelerometer (ADXL345; Analog Devices) as used in this study, indicates that a change of 1°C relative to 25°C could result in a 0.4 to 1.2 m*g* (1 *g* = 1,000 m*g*) change in acceleration value (1). It could therefore be hypothesized that the availability of temperature information alongside measurement of acceleration may aid the autocalibration process.

The current study aims to describe an autocalibration method that can be configured to take into account a potential temperature dependency of the sensor's response to acceleration. The second aim is to implement and evaluate the autocalibration method in a diverse sample of the global population. The third and final aim is to demonstrate the degree to which application of the autocalibration method has any significant impact on metrics derived for physical activity assessment.

## METHODS

### 

#### Population data.

The autocalibration method was evaluated based on data collected with wrist-worn raw accelerometry in subsamples of epidemiological cohorts from Africa, Europe, South America, and the Middle-East, representing locations with different gravity. Cohorts included: The Fenland Study (Cambridgeshire, UK) (21), a repeated cross-sectional survey of the Cameroon Physical Activity Study (3), the Kuwait Wellbeing Study, and the 1993 Pelotas birth cohort (Brazil) (28). The data subsamples in each cohort span most local seasons and represent very diverse populations, lifestyles, and environmental conditions. Basic cohort characteristics are described in Table 1.

**Table 1.** Cohort characteristics

Cohort	UK	Kuwait	Cameroon	Brazil
*n* (male/female)	407/514	72/48	144/167	100/100
Age, yr	50.3 (7.2)	43.0 (10.7)	40.3 (12.6)	18.4 (18–19)†
Weight, kg	77.1 (16.1)	81.8 (18.3)	76.8 (15.2)	65.8 (14.7)
Height, m	170.0 (9.6)	167.5 (8.5)	166.7 (8.4)	167.3 (8.3)
BMI, kg/m^2^	26.5 (4.6)	29.0 (5.3)	28.2 (9.6)	23.4 (4.7)
Monitor protocol, days	6	7	7	6
Sample frequency, Hz	60	50	100	85.7
Geographic latitude, °	52.2 N	29.4 N	5.1 N	31.8 S
Altitude, m	6	20	726/1,600‡	7
Magnitude of gravity, m·s^−2^*	9.8127	9.7928	9.7807	9.7947
Difference in gravity relative to UK, m*g*	0.0	−2.0	−3.3	−1.8
Seasonal distribution				
In Dec-Feb	23%	32%	47%	39%
In Mar-May	24%	27%	0%	10%
In Jun-Aug	30%	41%	13%	0%
In Sep-Nov	23%	1%	41%	50%

Data are expressed as mean (SD). BMI, body mass index.

* According to calculation with World Geodetic System 1984;

† age range;

‡ Yaounde and Bamenda.

The same accelerometer brand was used in all cohorts (GENEActiv; Activinsights, Kimbolton, UK). This accelerometer includes a triaxial acceleration sensor (ADXL345) with a ±8-*g* dynamic range and a 12-bit resolution and a temperature sensor (MCP9700T). Most of the devices used in the UK and Brazil cohort were older (lower serial number) than the devices used in the Kuwait and Cameroon cohorts. In all cohorts, participants were asked to wear the accelerometer on their nondominant wrist during sleeping and waking hour. All participants provided informed consent, and each study was approved by the local ethics committee.

#### Autocalibration method.

Two versions of the autocalibration method were designed and evaluated; one based on acceleration data only (C_1_) and one based on both acceleration and temperature (C_2_). For every consecutive time window of 10 s in a particular data record, the following signal features were extracted: average acceleration per axis, standard deviations in the acceleration per axis, and average temperature. For the calibration procedure, only time windows for which the standard deviation was <13 m*g* in all three axes were retained. Here, 13 m*g* was selected just above the empirically derived baseline (noise) standard deviation of 10 m*g* to retain only nonmovement periods. The resulting set of time windows, or calibration epochs, for each of the three axes can be presented in a three-dimensional space as an ellipsoid (22), an example of which is shown in Fig. 1. The deviation between 1 *g* and the Euclidean norm ($\sqrt{a_{x}^{2}+a_{y}^{2}+a_{z}^{2}}$) of the acceleration of the three axes is an indication of calibration error. Next, the axis-specific calibration for C_1_ can be defined as: *s*_*i*_^'^(*t*) = *d*_*i*_ + *s*_*i*_(*t*)·*a*_*i*_. Here, *s*_*i*_(*t*) and *s*_*i*_^'^(*t*) correspond to the acceleration signal before and after correction, respectively, *i* is the sensor axis (*x*, *y*, or *z*), *t* is the time point, *d*_*i*_ is the offset, and *a*_*i*_ is the gain factor. Six parameters are optimized for this model C_1_, while minimizing the average calibration error defined as absolute differences between 1 *g* and vector magnitudes (Euclidean norms) calculated across all calibration epochs. If temperature is taken into account (C_2_) the formula is: *s*_*i*_^'^(*t*) = *d*_*i*_ + *s*_*i*_(*t*)·*a*_*i*_ + [*T*(*t*) − *c*]·*m*_*i*_. Here, *T*(*t*) is the temperature at time point *t*, *c* is the average temperature in the ellipsoidal data as used for the autocalibration procedure, and *m*_*i*_ is the axis specific temperature-related offset corrections factor. The average temperature acts like a fixed reference point relative to which *d*_*i*_, *a*_*i*_, and *m*_*i*_ (9 parameters) are optimized. Mathematically, constant *c* could have been merged with *d*_*i*_ to shorten the equation but we have kept them separate to allow direct comparison of *d*_*i*_ parameters between the two autocalibration models.

An iterative closest point fitting process (ICP) of the moving average values to a sphere (C_1_) or a hypercylinder (C_2_) was used to optimize the six (C_1_) or nine (C_2_) calibration correction factors, respectively. Here, the hypercylinder corresponds to the 1-*g* sphere augmented with a linear temperature offset adjustment for C_2_. The procedure was followed by downweighting outliers to minimize the impact of nonstationary data not being excluded based on the 13-m*g* threshold as mentioned earlier. Here, the weighting was calculated as 1-*g* divided by the absolute difference between the Euclidean norm corresponding to a 10-s calibration window (1 point on the ellipsoid) and 1 *g* with 100 being the maximum weighting. Consequently, all data points with <10-m*g* calibration error had a weighting of 100 (1 *g*/0.01 *g* = 100). The weighting was updated at every stage of the iterative process. The ICP starting points were chosen based on the assumption that the optimal calibration factors is the set representing the local minimum error nearest to perfect factory calibration, with *d*_*i*_ = 0, *a*_*i*_ = 1, and *m*_i_ = 0. The ICP was limited to a maximum of 1,000 iterations and terminated sooner if iterative change in error was <1^−10^ *g*.

To ensure a meaningful and robust autocalibration, it was only executed when the calibration ellipsoid was sufficiently sparsely populated with data points (calibration epochs). For this evaluation, we used a sparseness criteria of at least one ellipsoid value higher than 300 m*g* and at least one value lower than −300 m*g* for each of the three sensor axes. Only measurements were considered that lasted for at least 24 h as shorter measurements are commonly excluded when assessing habitual physical activity during free-living conditions.

To minimize signal processing time, the autocalibration method initially only uses the first 72 h (3 days) of a measurement file based on which calibration error reduction is evaluated. If the file length is <3 days, then all available data are used. If calibration error is not reduced to <10 m*g* or if the ±300-m*g* criteria for ellipsoid data sparseness is not met, additional chunks of 12-h data are iteratively added until either error and sparseness criteria are met or until the end of the file is reached. The criterion of 10 m*g* was considered close to the resolution of the data (3.9 m*g*) and a realistic target based on pilot tests. Calibration error below the sensor resolution is theoretically possible, but these calibration errors may not be distinguishable from the impact of data resolution boundaries. Therefore, a calibration error reduction to <10 m*g* was considered acceptable. If the calibration error after autocalibration was higher than before autocalibration, then correction factors were replaced by default values 1 and 0 for gain and offset, respectively. The latter was done to avoid a negative influence of autocalibration on the data.

The method has been released as function *g.calibrate* in R-package *GGIR*, which currently works with binary data collected with the accelerometer used in the current study as well as its predecessor, GENEA (14). Additionally, an extract of the R-code related to the ICP fitting process is provided in the appendix.

#### Evaluation.

The absolute difference between 1 *g* and the Euclidean norm of the values of the three axes was averaged per measurement file (1 file = 1 participant) and used as an indicator of calibration error before autocalibration (C_0_), following autocalibration without temperature compensation (C_1_), and following autocalibration with temperature compensation (C_2_).

Further, we assessed the impact of autocalibration on population estimates of physical activity using two commonly used metrics of body movement: the Euclidean Norm Minus One with negative values rounded up to zero (ENMO) and band-pass filtering of three axis followed by Euclidean Norm of the resulting signals (BFEN), as previously described (11, 13, 25). BFEN was applied with a fourth order band-pass Butterworth filter with cut-off frequencies 0.5 and 15 Hz. Metric ENMO is similar in design compared with a metric used by colleagues, named SVMgs (7, 23, 29). See Hildebrand et al. (15) for a discussion on the subtle differences between SVMgs and ENMO.

Here, we looked at the average metric output and its distribution over each participant's measurement record based on 5-s epoch averages. The impact on the distribution was quantified as changes to the 5th, 25th, 50th, 75th, 95th, and 97.92nd percentiles. The latter percentile (97.92) corresponds to the 30 most active minutes in a day. All participant-level values were summarized as mean (SD) across each cohort. Data cleaning stages, including nonwear detection, were applied as reported previously (11, 14, 24). Detected monitor wear duration was used to evaluate whether monitor wear duration plays a role in the success of the autocalibration procedure.

Finally, to estimate the relative importance of correcting offset or gain factors we selected a random sample of 20 accelerometer recordings from the Pelotas cohort and investigated how autocalibration performance is affected when optimizing only offset or gain, with the corresponding other set of factors fixed to 1 (gain) or 0 *g* (offset), respectively.

#### Statistics.

All statistical analyses were conducted in R (http://cran.r-project.org/). Wilk's lambda test was used to compare the three autocalibration configurations across all percentiles. If Wilk's lambda test indicated a significant difference, then repeated measures ANOVA was used to compare the three autocalibration configurations per metric, using the function *lme* from the *nlme-*package and the function *anova* from the *stats*-package (20a). Post hoc pair-wise Tukey tests were performed using the function *glht* from the *multcomp* package (16). Significance was set at *P* < 0.05.

## RESULTS

Average calibration correction factors are reported in Table 2. Application of the autocalibration method significantly reduced calibration error in all cohorts (*P* < 0.01), with improvements being greatest in the Brazilian cohort (from 76.7 to 8.0 m*g*) and smallest in the Kuwaiti cohort (from 16.6 to 3.0 m*g*; see Table 3). However, no significant further reduction in calibration error was observed in any of the four cohorts between autocalibration with temperature utilization (C_2_), compared with that without temperature utilization (C_1_) (*P* > 0.05; see Table 3). The percentage of files with calibration error under 10 m*g* was 6.1, 94.4, and 99.0% for C_0_, C_1_, and C_2_ respectively (Pearson's Chi-squared: χ^2^ = 3,816.0, df = 2, *P* < 0.0001). An animation of the calibration ellipsoid before and after calibration can be found on our website: http://www.mrc-epid.cam.ac.uk/research/resources.

**Table 2.** Average calibration correction factors

Location/Correction Factor	*x*	*y*	*z*
UK			
*a*	0.99824 (0.0046)	0.99777 (0.01079)	1.00133 (0.01068)
*d*	−0.00738 (0.00851)	−0.00494 (0.0164)	−0.01177 (0.03719)
*m*	−0.00001 (0.00083)	0.00022 (0.00128)	0.00392 (0.00134)
Kuwait			
*a*	1.00453 (0.00295)	1.0001 (0.00404)	1.00400 (0.00685)
*d*	−0.00124 (0.00280)	0.00042 (0.00303)	0.02321 (0.01380)
*m*	0.00005 (0.00049)	0.00031 (0.00062)	0.00101 (0.00081)
Cameroon			
*a*	1.00285 (0.00223)	0.99729 (0.00477)	1.00437 (0.00247)
*d*	0.00987 (0.00725)	0.00862 (0.00921)	0.07145 (0.02686)
*m*	−0.00009 (0.00093)	0.00103 (0.00142)	0.00179 (0.00142)
Brazil			
*a*	0.99953 (0.00756)	0.98992 (0.01386)	1.00356 (0.01198)
*d*	0.02570 (0.02217)	0.01010 (0.02360)	0.10545 (0.03534)
*m*	0.00001 (0.00169)	0.00067 (0.00231)	0.00365 (0.00106)

Data are expressed as mean (SD). *d*, offset (*g*); *a*, gain; *m*, temperature-dependent offset (*g*/°C).

**Table 3.** Calibration error across study locations

	UK	Kuwait	Cameroon	Brazil
*N*	921	120	311	200
Calibration error, m*g*: C_0_	25.7 (13.9)	16.6 (7.1)	47.3 (16.3)	76.7 (24.0)
Calibration error, m*g*: C_1_	7.4 (2.9)	3.0 (0.7)	3.0 (1.2)	8.0 (2.3)
Calibration error, m*g*: C_2_	4.9 (2.0)	2.5 (0.6)	2.7 (1.1)	5.3 (1.5)
*F* value, *P* < 0.001 for all	2,022*	375*	2,286*	1,756*
*n* with error >10 m*g* (C_0_; C_1_; C_2_)	862; 63; 16	94; 0; 0	301; 0; 0	200; 24; 0
*n* with <72 h (3 days) of wear	24	10	4	6

C_0_, no autocalibration; C_1_, autocalibration without temperature utilization; C_2_, autocalibration with temperature utilization.

* Significant pair-wise difference between C_0_–C_1_ and C_0_–C_2_ (Tukey test, *P* < 0.001). No significant difference was observed between C_1_ and C_2 _(Tukey test, *P* > 0.05).

Application of the autocalibration method had a significant impact on the average and distribution of acceleration metric output in each of the four cohorts (*F* > 5.8, *P* < 0.001). The magnitude of the difference between C_0_ and C_1_ for metric BFEN was systematically <1 m*g*, which was in contrast to metric ENMO for which differences of 20 m*g* and higher were observed between C_0_ and C_1_ (see Tables 4 and 5). Post hoc Tukey analyses revealed no significant difference in metric output between C_1_ and C_2_, except for the lower range in the distribution of acceleration values in the UK cohort, see Tables 4 and 5.

**Table 4.** Impact of autocalibration on daily wrist acceleration calculated with metric ENMO

Cohort/Metric	C_0_	C_1_	C_2_	*P* Value*	
UK					
Daily average	34.4 (8.4)	31.8 (11.8)	31.3 (8.3)	<0.001	●
P5	6.2 (6.9)	4.5 (1.7)	3.6 (1.8)	<0.001	■
P25	13.7 (9.9)	9.4 (3.3)	7.7 (3.5)		■
P50	27.1 (11.6)	24 (7.4)	23.7 (7.6)		●
P75	46.4 (14.8)	44.5 (12.2)	44.6 (12.3)		●
P95	87.4 (29.6)	86.1 (28.4)	86.5 (28.4)		●
P97.92	113.9 (49)	112.7 (48)	113 (48)		●
Kuwait					
Daily average	28.6 (8.1)	24.6 (9.3)	24.5 (8.1)	<0.001	●
P5	5.7 (4.2)	2.8 (1.0)	2.7 (0.9)	<0.001	●
P25	12 (6.2)	6.3 (2.6)	6.1 (2.6)		●
P50	21.6 (7.8)	17.3 (6.1)	17.3 (6.1)		●
P75	36.4 (11.5)	33.2 (10.6)	33.2 (10.6)		●
P95	74.4 (36)	72.2 (35.9)	72.1 (36.0)		●
P97.92	100.9 (66.4)	99 (66.5)	98.9 (66.6)		●
Cameroon					
Daily average	53.3 (16.4)	34.5 (18.8)	34.5 (16.4)	<0.001	●
P5	18.1 (8.5)	3.6 (0.9)	3.5 (0.9)	<0.001	●
P25	32.4 (11.1)	8.4 (3.6)	8.3 (3.7)		●
P50	45.9 (12.4)	25.3 (7.5)	25.3 (7.6)		●
P75	65.8 (29.3)	48.8 (28.6)	48.8 (28.5)		●
P95	112.8 (71.6)	98.9 (72.4)	99 (72.3)		●
P97.92	143.7 (93.1)	130.7 (93.6)	130.7 (93.6)		●
Brazil					
Daily average	80.6 (12.5)	39.7 (19.7)	39.5 (12.4)	<0.001	●
P5	33.6 (15.3)	4.6 (1.7)	3.7 (1.6)	<0.001	●
P25	55.2 (18.6)	11.6 (5.4)	10.4 (5.5)		●
P50	74.2 (19.7)	29 (10.7)	28.8 (11.0)		●
P75	96.6 (22.5)	54.3 (17.4)	54.7 (17.8)		●
P95	148.5 (37.7)	111.1 (37.5)	111.8 (37.4)		●
P97.92	183.4 (55)	147.7 (55.7)	148.5 (55.7)		●

Data are presented as sample mean (SD) and percentiles based on 5-s epoch averages; *Pk* = *k*th percentile. ENMO (in m*g*), the Euclidean Norm Minus One; C_0_, no autocalibration; C_1_, autocalibration without temperature; C_2_, autocalibration with temperature.

* *P* value for ANOVA and Wilk's lambda; *P* values for Tukey test are indicated with the following symbols: ●, significant pair-wise differences between C_0_–C_1_ and C_0_–C_2_; ▲, significant pair-wise differences for C_0_–C_1_ and C_1_–C_2_; ■, significant pair-wise difference for C_0_–C_1_, C_0_–C_2_, and C_1_–C_2_.

**Table 5.** Impact of autocalibration on daily wrist acceleration calculated with metric BFEN

Cohort/Metric	C_0_	C_1_	C_2_	*P* Value*	
UK					
Daily average	122.7 (25.5)	122.5 (25.5)	122.6 (25.5)	<0.001	●
P5	10.5 (1.9)	10.5 (1.9)	10.5 (1.9)	<0.001	▲
P25	25 (15.0)	24.9 (14.9)	25 (14.9)		●
P50	113.6 (31.3)	113.5 (31.2)	113.5 (31.2)		●
P75	189.4 (42.5)	189.2 (42.4)	189.2 (42.4)		●
P95	295.7 (57.5)	295.3 (57.5)	295.4 (57.5)		●
P97.92	344.4 (73.7)	344 (73.6)	344.1 (73.7)		●
Kuwait					
Daily average	104.4 (23.5)	104.7 (23.5)	104.7 (23.5)	<0.001	●
P5	9.1 (3.1)	9.1 (3.1)	9.1 (3.1)	<0.001	●
P25	28.7 (17.4)	28.8 (17.5)	28.8 (17.5)		●
P50	91.2 (27.3)	91.4 (27.4)	91.4 (27.4)		●
P75	155 (37.5)	155.4 (37.5)	155.4 (37.5)		●
P95	257 (62.4)	257.7 (62.4)	257.7 (62.4)		●
P97.92	306.3 (77.7)	307.1 (77.7)	307.1 (77.7)		●
Cameroon					
Daily average	125.6 (24.4)	125.9 (24.3)	125.8 (24.4)	<0.001	●
P5	8.7 (3.9)	8.7 (3.9)	8.7 (3.9)	<0.001	●
P25	39.2 (19.6)	39.3 (19.6)	39.3 (19.6)		●
P50	118 (27.9)	118.2 (27.9)	118.2 (27.9)		●
P75	187.6 (36.6)	188 (36.7)	187.9 (36.7)		●
P95	294.2 (62.4)	294.7 (62.6)	294.7 (62.5)		●
P97.92	346.5 (83.0)	347.2 (83.1)	347.1 (83.1)		●
Brazil					
Daily average	138.5 (31.1)	138.2 (31.1)	138.2 (31.1)	<0.001	●
P5	10.1 (5.3)	10.2 (5.3)	10.1 (5.3)	<0.001	ns
P25	42.1 (27.1)	42.1 (27.1)	42.1 (27.1)		●
P50	126.8 (38.3)	126.6 (38.3)	126.6 (38.3)		●
P75	208.6 (44.5)	208.1 (44.6)	208.2 (44.5)		●
P95	328.3 (58.3)	327.5 (58.0)	327.6 (58.2)		●
P97.92	383.6 (71.5)	382.6 (71.2)	382.7 (71.4)		●

Data are presented as sample mean (SD) and percentiles based on 5-s epoch averages; *Pk* = *k*th percentile. BFEN (in m*g*), band-pass filtering of t3 axis followed by Euclidean Norm of the resulting signals. C_0_, no autocalibration; C_1_, autocalibration without temperature; C_2_, autocalibration with temperature;

* *P* value for ANOVA and Wilk's lambda; *P* values for Tukey-test are indicated with the following symbols: ●, significant pair-wise differences between C_0_–C_1_ and C_0_–C_2_; ▲, significant pair-wise differences for C_0_–C_1_ and C_1_–C_2_; ■, significant pair-wise difference for C_0_–C_1_, C_0_–C_2_, and C_1_–C_2_; ns, *P* value for ANOVA >0.05.

The minimum within-person temperature range observed within the ellipsoid data was 8.8, 9.7, 6.4, and 7.1°C for UK, Kuwait, Cameroon, and Brazil, respectively.

For the evaluation of the relative importance of offset and gain (Pelotas subset), autocalibration based on only offset correction or only gain correction reduced a 65.0 ± 26.7 m*g* calibration error (C_0_) to 12.4 ± 12.7 and 51.8 ± 31.2 m*g*, respectively. Additional ultization of temperature reduced these calibration errors to 10.2 ± 13.3 and 45.0 ± 23.9 m*g*, respectively, while optimizing both offset and gain resulted in calibration errors of 4.6 ± 1.3 and 7.7 ± 2.7 m*g* for C_2_ and C_1_, respectively.

## DISCUSSION

The autocalibration method as presented allows for a significant reduction in average calibration error under a wide range of study conditions. Temperature ultization did not result in a significant further reduction of average calibration error for the measures selected. However, inspection of the derived temperature offset correction factors (Table 2) indicates that temperature ultization could be essential for sections of the signal with temperature conditions far away from the average temperature. For example, in the UK cohort the average temperature offset correction factor for the *z*-axis was 0.00392 (Table 2), which given a temperature difference of 5°C would result in a change of acceleration of 0.0196 *g* (5 × 0.00392 *g*). A value of 19.6 m*g* may be considered high in the context of the acceleration value distribution as provided in Tables 4 and 5. The significant difference as found between C_1_ and C_2_ in the lower end of the metric value distribution in the UK cohort hints at an impact of temperature ultization that will only be visible in the most inactive parts of a day. Considering that sleep is likely to take up >25% of a day, it seems unlikely that the temperature dependency of the 5th and 25th percentiles relates to wake-time behavior. Instead, accounting for temperature dependency may help to improve estimates of monitor nonwear time and the detection of sleep stages in future research.

The implementation of the autocalibration method had a significant impact on the average and distribution of metric outputs; however, substantially more so for metric ENMO compared with metric BFEN. In our previous study we observed that metrics ENMO and BFEN are highly correlated but not identical (11). Metric ENMO may be more appropriate for energy expenditure estimation and easier for researchers to describe, replicate, and interpret (11). In addition, the frequency filtering as part of metric BFEN effectively reduces calibration offset error, which explains why the autocalibration procedure as evaluated here shows only minor impact on these estimates. Temperature changes tend to be slow, which the band-pass filter would catch and remove as low frequency components (11). We conclude from this that autocalibration will have an important impact on studies that rely on average and distribution characteristics of metric ENMO but much less so for metric BFEN. Note that these findings should not be confused for the validity of metric BFEN or ENMO.

The strong relative importance of offset correction as seen in the subsample of 20 individuals combined with the fairly constant absolute difference between the cohort percentiles corresponding to C_0_ and to C_1_ (Tables 4 and 5) indicates that the offset calibration has a bigger impact compared with gain calibration. Translating this observation to physical activity research means that the impact of calibration error and therefore the benefit of autocalibration will be relatively high for physical activities involving low acceleration and relatively low for activities involving high magnitude accelerations.

Results indicate that the autocalibration method works under a wide range of experimental conditions, spanning different geographical latitudes, different seasons affecting temperature variation during the day, different populations affecting movement and activity patterns, different built environments, and different adult age groups. Nonetheless, the dataset as presented is insufficient to investigate the causal relationship between specific study conditions and calibration error. The difference in the precision gain of autocalibration between UK and Brazil on the one hand and Cameroon and Kuwait on the other hand may indicate that the relatively newer devices used for Cameroon and Kuwait have less calibration error. Again, a lack of standardized conditions complicates this comparison. It is also important to note that the proposed method effectively expresses all data relative to local gravity that has known geographical variation; one would need to multiply with the magnitude of local gravity to convert to absolute acceleration in meters per second squared. Despite the challenges in directly comparing the four cohorts, the results stratified by cohort illustrate that the method succeeds in reducing error in each of the four study settings and with an impact on typical physical activity summary measures proportional to baseline calibration error (C_0_).

The current study was done with wrist-worn accelerometers. Compared with other body locations wrist attachment may allow for easier collection of sparse ellipsoidal data and by that enhancing the autocalibration process. Therefore, caution is needed when implementing this method on data collected from other body locations.

In conclusion, the autocalibration method as presented reduces the calibration error in acceleration data from wrist-worn sensors as collected on four continents. Temperature ultization seems essential for those sections of the signal where temperature deviates substantially from the average temperature, but less so for overall summary measures related to the average and distribution of the magnitude of acceleration over several days.

## GRANTS

This research was supported by funding from the Wellcome Trust, the Medical Research Council (MC_UU_12015/3), and His Highness Shiekh Nasser Al-Mohammad Al-Sabah, and Dasman Diabetes Institute.

## DISCLOSURES

Joss Langford is employed by Activinsights We declare that we have no competing interests.

## AUTHOR CONTRIBUTIONS

Author contributions: V.T.v.H., Z.F., J.L., and S.B. conception and design of research; V.T.v.H. analyzed data; V.T.v.H. and S.B. interpreted results of experiments; V.T.v.H. and T.W. prepared figures; V.T.v.H. drafted manuscript; V.T.v.H. and S.B. edited and revised manuscript; V.T.v.H., Z.F., J.L., F.A., A.M., I.C.d.S., M.I.T., T.W., N.J.W., and S.B. approved final version of manuscript; F.A., A.M., I.C.d.S., and N.J.W. performed experiments.

## Appendix I: EXTRACT OF R-CODE RELATED TO ICP PROCEDURE FROM R-PACKAGE GGIR

The variable “input” is the average acceleration per axis per epoch provided as a matrix with three columns corresponding to the three axis. Variable “inputtemp” is the average temperature per epoch provided as a matrix with the temperature values replicated in three columns.

meantemp = mean(as.numeric(inputtemp[,1]))

inputtemp = inputtemp - meantemp

translate = rep(0, ncol(input)); gain = rep(1, ncol(input))

tempoffset = rep(0, ncol(input))

weights = rep(1, nrow(input))

res = Inf

maxiter = 1000

tol = 1e-10

for (iter in 1:maxiter) {

curr = gain(input, center = -translate, gain = 1/gain) +

gain(inputtemp, center = F, gain = 1/tempoffset)

closestpoint = curr/sqrt(rowSums(curr^2))

k = 1

translatech = rep(0, ncol(input)); gainch = rep(1,ncol(input))

toffch = rep(0, ncol(inputtemp))

for (k in 1:ncol(input)){

fobj = lm.wfit(cbind(1, curr[,k],inputtemp[,k]),

closestpoint[,k, drop = F], w = weights)

if (use.offset == TRUE) translatech[k] = fobj$coef[1]

if (use.gain == TRUE) gainch[k] = fobj$coef[2]

if (use.temp == TRUE) toffch[k] = fobj$coeff[3]

curr[,k] = fobj$fitted.values

}

translate = translate + translatech / (gain * gainch)

if (use.temp == TRUE) tempoffset = tempoffset * gainch + toffch

gain = gain * gainch

res = c(res, 3 * mean(weights*(curr-closestpoint)^2/sum(weights)))

weights = pmin(1/sqrt(rowSums((curr - closestpoint)^2)), 1/0.01)

if (abs(res[iter+1] - res[iter]) < tol) break
